# Anterior cruciate ligament reconstruction in a patient with Athetoid cerebral palsy: a case report

**DOI:** 10.1186/1758-2555-4-36

**Published:** 2012-10-02

**Authors:** Takuya Tajima, Etsuo Chosa, Keitarou Yamamoto, Katsuhiro Kawahara, Nami Yamaguchi, Shinji Watanabe

**Affiliations:** 1 Division of Orthopedic Surgery, Department of Medicine of Sensory and Motor Organs, Faculty of Medicine, University of Miyazaki, 5200 Kihara, Kiyotake, Miyazaki 889-1692, Japan

**Keywords:** Athetoid cerebral palsy, Anterior cruciate ligament reconstruction, Involuntary movement, Stress radiography

## Abstract

Recent years have seen ACL reconstruction performed in a broad range of patients, regardless of age, sex or occupation, thanks to great advances in surgical techniques, instrumentation and the basic research. Favorable results have been reported; however, we have not been able to locate any reports describing ACL reconstruction in patients with athetoid cerebral palsy.

We present herein a previously unreported anterior cruciate ligament (ACL) reconstruction performed in a patient with athetoid cerebral palsy. The patient was a 25-year-old woman with level II athetoid cerebral palsy according to the Gross Motor Function Classification System. She initially injured her right knee after falling off a bicycle. Two years later, she again experienced right-knee pain and a feeling of instability. A right-knee ACL tear and avulsion fracture was diagnosed upon physical examination and confirmed with magnetic resonance imaging (MRI) and X-ray examination at that time. An ACL reconstruction using an autologous hamstring double-bundle graft was performed for recurrent instability nine years after the initial injury. Cast immobilization was provided for 3 weeks following surgery and knee extension was restricted for 3 months with the functional ACL brace to prevent hyperextension due to involuntary movement. Partial weight-bearing was started 1 week postoperatively, with full weight-bearing after 4 weeks. The anterior drawer stress radiography showed a 63% anterior displacement of the involved tibia on the femur six months following the surgery, while the contralateral knee demonstrated a 60% anterior displacement of the tibia. The functional ACL functional brace was then removed. A second-look arthroscopy was performed 13 months after the ACL reconstruction, and both the anteromedial and posterolateral bundles were in excellent position as per Kondo’s criteria. The Lachman and pivot shift test performed under anesthesia were also negative. An anterior drawer stress radiography of the involved knee at 36 months following surgery showed a 61% anterior translation of the tibia. The preoperative symptoms of instability resolved and the patient expressed a high degree of satisfaction with the result of her surgery.

## Background

Anterior cruciate ligament (ACL) injury is common with sporting activity. ACL laxity sometimes causes knee joint instability in activities such as cutting or pivoting, which can lead to articular cartilage degradation and/or meniscus injury [1-3].

Recent years have seen ACL reconstruction performed in a broad range of patients, regardless of age, sex or occupation, thanks to great advances in surgical techniques, instrumentation and the basic research. Favorable results have been reported for many [4-8]. We have been unable to identify any reports describing ACL reconstruction in patients with athetoid cerebral palsy in the literature. We are presenting our experience with ACL reconstruction in such a patient. The patient and her family were informed that data concerning the case would be submitted for publication, and gave their consent. With the patient`s agreement, we are reporting on the case with its clinical features and the treatment.

## Case presentation

**Figure 1:**
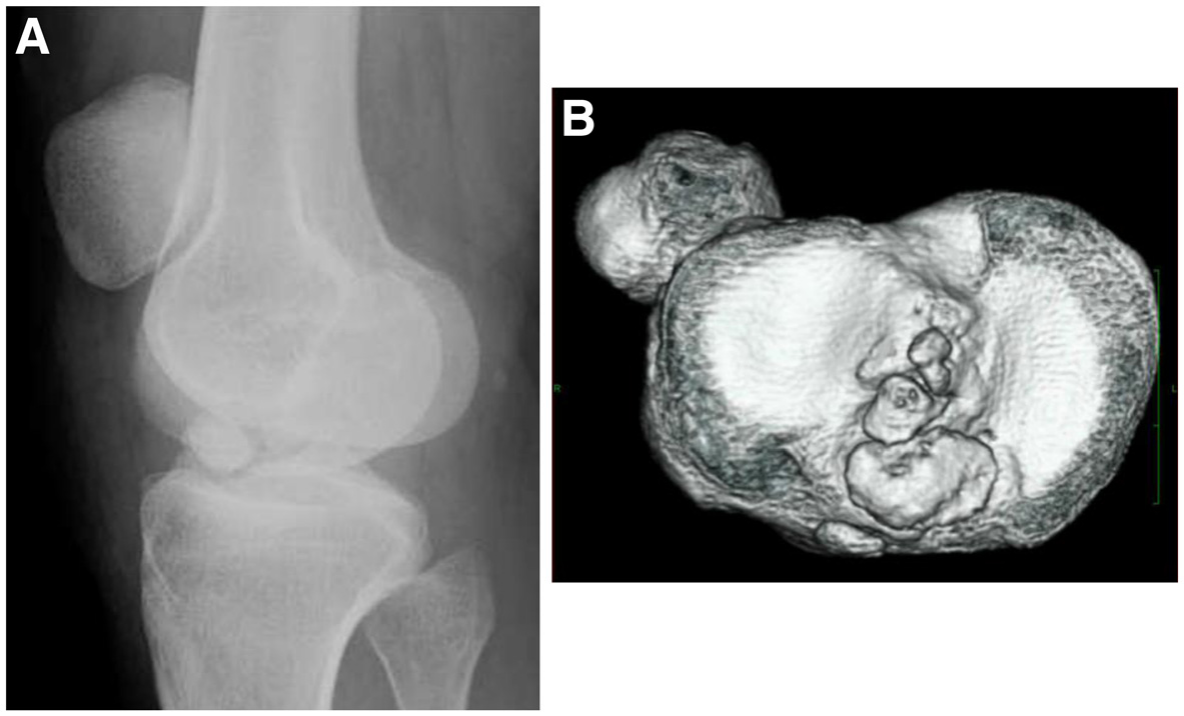
**X-ray (A) and CT findings (B). **The round-shaped intraarticular bone fragment was observed on X-ray (**A**). Three bone fragments were confirmed on CT (**B**).

**Figure 2:**
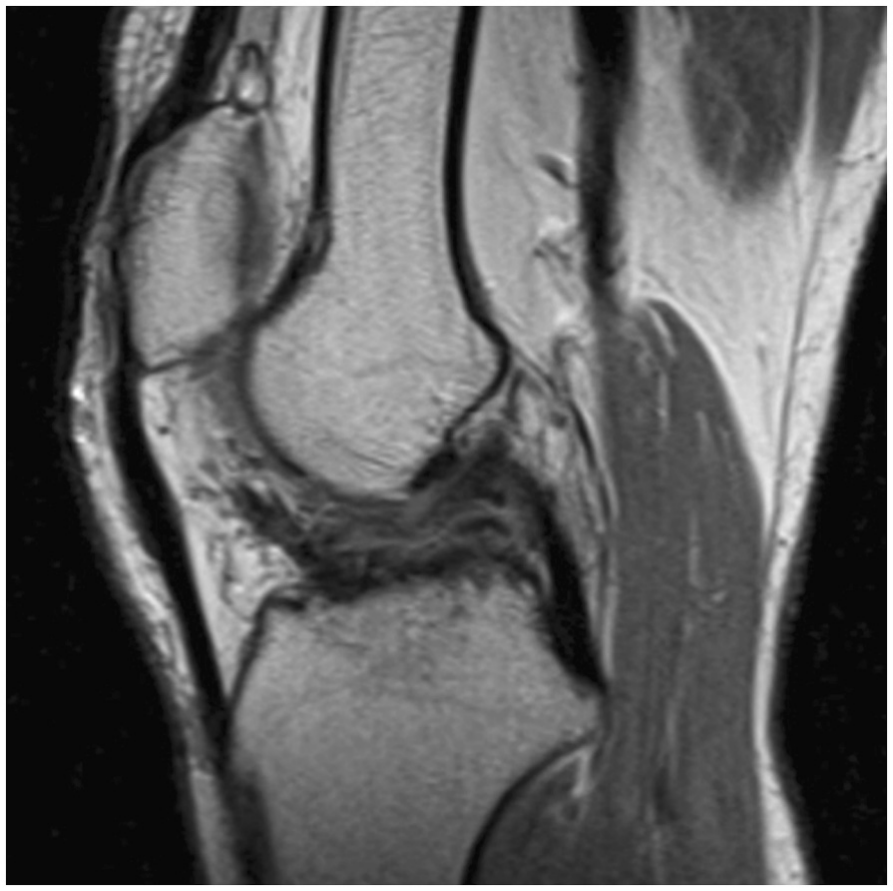
**MRI findings. **Sagittal fast-spin-echo magnetic resonance imaging shows the disappearance of the distal ACL attachment.

The patient is a 25-year-old Japanese woman afflicted with athetoid cerebral palsy as a result of a six minutes period of anoxia at the time of birth. Both upper and lower limbs exhibited pronounced involuntary movements and she was rated as level II according to the Gross Motor Function Classification System (GMFCS) [9]. She presented with a combined crouch and spastic gait pattern. Both hips internally rotated during gait with an equinus foot position, however, she was able to ambulate and ride a bicycle without assistance. She was able to climb stairs one step at a time. She initially twisted and injured the right knee in a fall from a bicycle in 1999. The obvious injury was not pointed out in X-ray examination by the local physician, and the injury was left untreated at that time. She reported an uncomfortable sensation in her right knee since her fall in 1999. She again experienced pain and instability in the right knee while walking and using the stairs in 2001. X-ray, CT and physical examination by another local physician diagnosed an avulsion fracture involving the ACL at the tibial insertion with an intraarticular loose body (Figure 1A and 1B). Arthroscopic resection of the loose body and fixation of the avulsed bone fragment under pull-out technique was performed in 2001. Bone healing at the ACL tibial insertion site was not successful and symptoms still remained. An additional surgery for resection of the bone fragment at the tibial ACL insertion was performed in 2003. The patient continued to feel pain and instability when walking after the second surgery, even with the use of the functional ACL brace. Our examination revealed physical findings of involuntary movement, making objective evaluation of ACL function difficult to perform using accurate testing or instruments. Her involuntary movement was increased when she was nervous, such that attaching instrumentation around her leg for testing created anxiety and therefore exaggerated her involuntary movement. Her Lysholm score was 31 points and an International Knee Documentation Committee (IKDC) score was 26.4 [10]. In addition, she felt a strong sense of discomfort when walking, and the visual analog scale (VAS) was 66 mm on a 100-mm scale. Her popliteal angle was 0°, and no voluntary hamstring contraction was palpated. The patient was able to ambulate using the functional ACL brace. Magnetic resonance imaging (MRI) under sedation indicated that the distal insertion of the ACL was not clear (Figure 2). A degenerative change in the posterior horn of the lateral meniscus was also confirmed on MRI.

**Figure 3:**
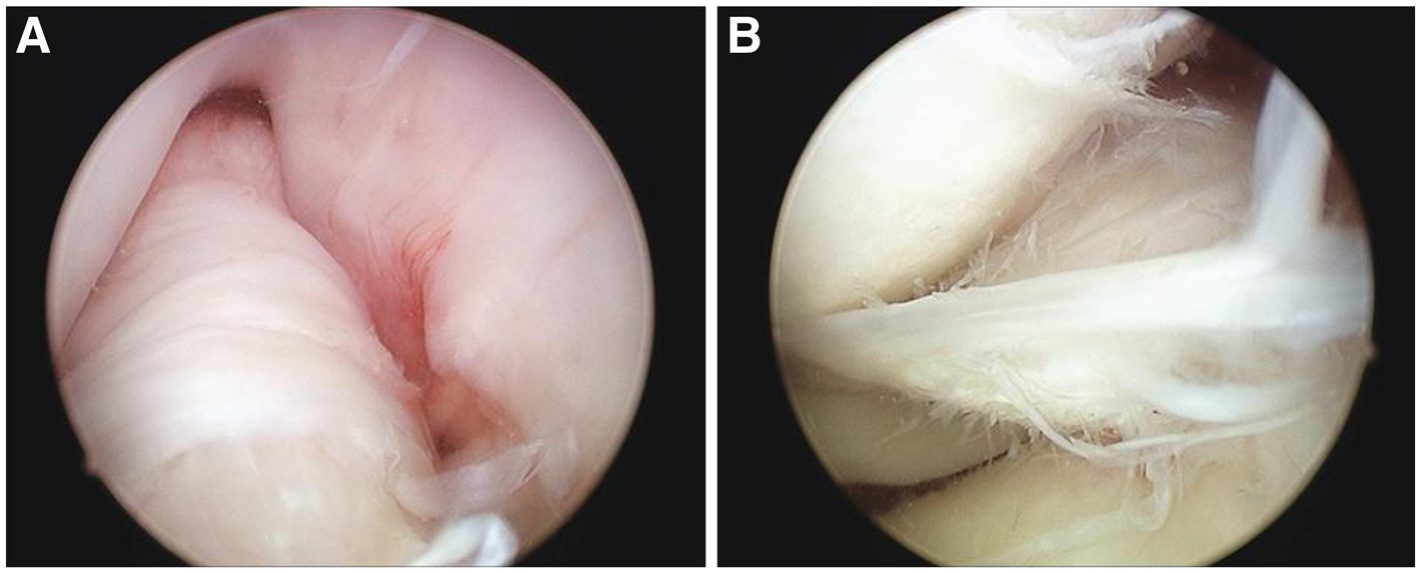
**Arthroscopic findings (A and B). **Fibrous changes were observed around the ACL remnant, and loss of ACL tension was also observed (**A**). The posterior horn of the lateral meniscus presented with a degenerative tear and flap (**B**).

**Figure 4:**
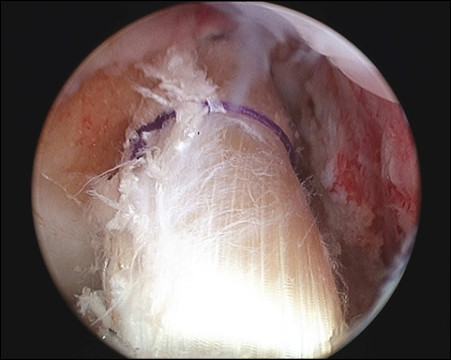
**ACL reconstruction. **Arthroscopic findings of the anatomical double bundle autologous hamstring graft ACL reconstruction.

The clinical manual tests for ACL laxity, to include the Lachman and Pivot shift tests had positive findings under anesthesia. Examination using the arthrometer such as Kneelax or KT-2000 was not performed. Arthroscopy revealed the presence of tiny bone fragments at the site of the ACL-tibial attachment, with reduced ACL tension and degenerative tearing of the posterior portion of the lateral meniscus (Figure 3A and 3B). Anatomical double-bundle autologous hamstring graft ACL reconstruction and partial menisectomy was performed. Articular cartilage degeneration was noted on the lateral femoral condyle and was classified as Grade II according to the Outerbridge classification. The semitendinosus and gracilis tendons were each double folded to create an anteromedial bundle with a diameter of 7.0 mm and a posterolateral bundle with a diameter of 5.5 mm (Figure 4). Using an accessory far medial portal, the respective bone tunnels were created at the anatomical positions proposed by Yasuda *et al.*[11]. The bundles were fixed with the knee at 20° of flexion using an EndoButton CL (Smith & Nephew, Andover, MA) on the femoral side, and a double-spike plate (Meira, Aichi, Japan) on the tibial side.

**Figure 5:**
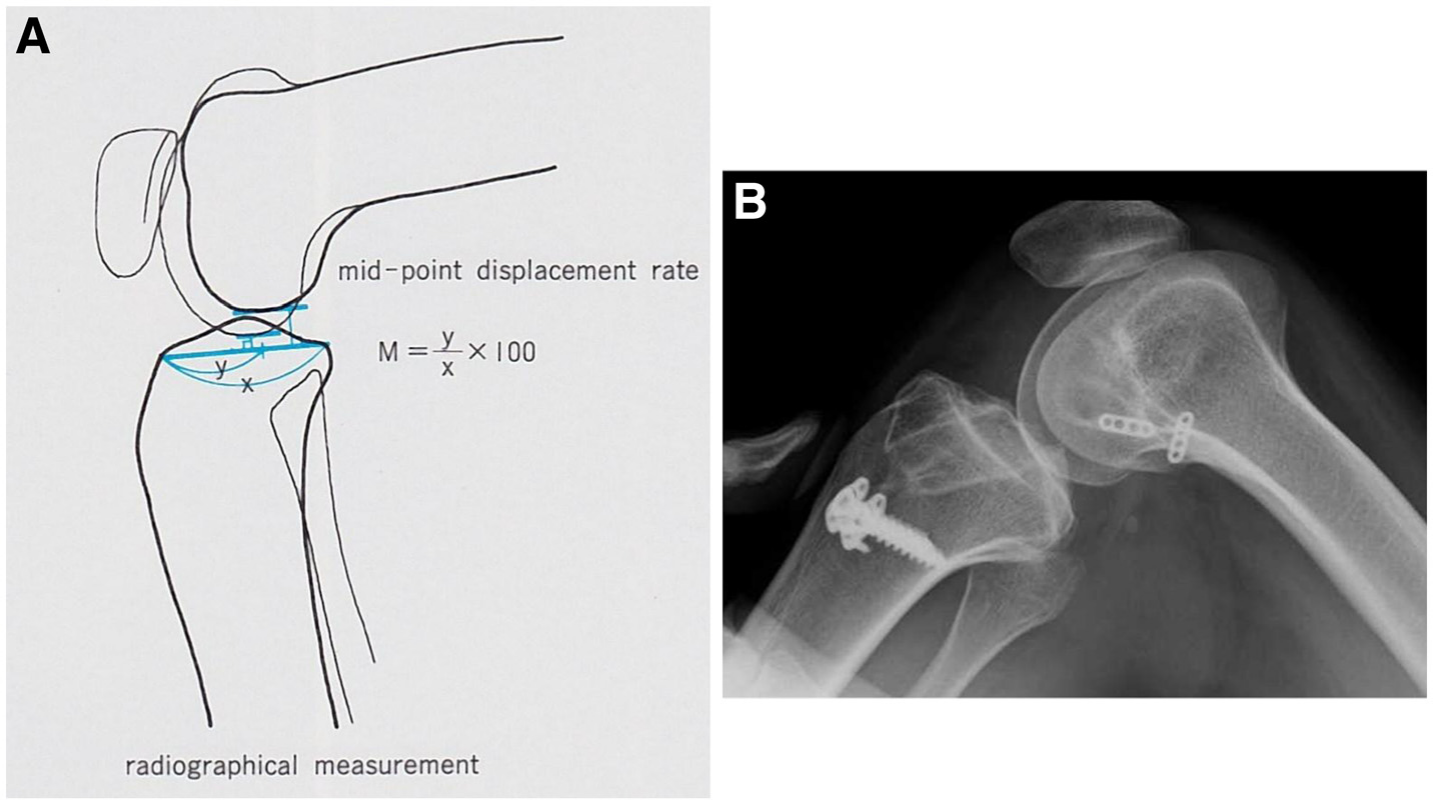
**Stress X-ray findings. **Stress radiography evaluated based on the results of midpoint measurements (**A**). A finding of greater than 70% of anterior tibial translation indicates ACL function failure. The results of the present case, at the six months postoperative evaluation revealed a 63% anterior tibial translation (**B**).

Cast immobilization with the knee at 20° of flexion was utilized for the first three postoperative weeks to prevent hyperextension due to involuntary movement. Knee extension was restricted to −10° for the first 3 months using the functional ACL brace. Partial weight-bearing was started at 1 week following surgery, with full weight-bearing at 4 weeks. Involuntary leg movements made an effective strengthening protocol impossible to perform, so the functional ACL brace was kept in place for 6 months following surgery. Isokinetic strength measurements were also not taken due to her involuntary movement. In addition, accurate assessment with manual examination techniques, such as the Lachman test, or objective measurements of joint stability with an arthrometer were also omitted due to her involuntary movements. Postoperative evaluations were therefore performed using stress radiography with the knee at 90° of flexion, pulling the tibia forward, to evaluate the anterior displacement rate of the tibia based on the results of midpoint measurements (Figure 5A) [12]. Stress radiograph evaluation at 6 months following surgery showed a 63% anterior displacement of the tibia. This finding indicated that the reconstructed ACL had appropriate available tension (Figure 5B), so the knee brace was removed. The stress radiography of the contralateral, non-injured, knee showed a similar 60% anterior translation of the tibia (data not shown). Moreover, preoperative knee instability had disappeared, and the patient expressed a high degree of satisfaction with the postoperative results. The patient felt no knee pain or instability during the course of activities of daily living or even when walking quickly at 8 months after the operation.

Examination of the patient 1 year after surgery revealed a Lysholm score of 72 points and an IKDC score of 64.4. The patient continued to feel no knee pain or instability with activities of daily living or when walking quickly. VAS evaluation was scored at 0 mm, with absolutely no pain or sense of discomfort. She did have tenderness upon palpation of the tibial fixation devices.

**Figure 6:**
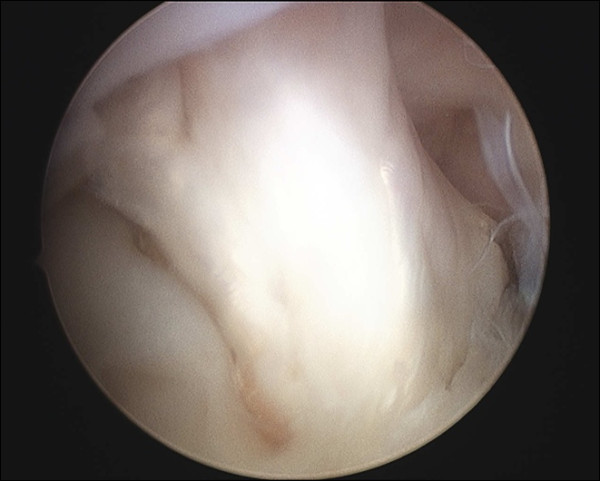
**Second-look arthroscopic findings. **Thirteen-month postoperative second-look arthroscopic findings. Thirteen months following ACL reconstruction, the second-look arthroscopic findings indicated that the reconstructed ACL, both anteromedial and posterolateral bundles, shows excellent results in terms of graft thickness, apparent tension and synovium coverage.

A second-look arthroscopic evaluation was performed 13 months following the ACL surgery and removal of the fixation devices. The Lachman and pivot shift tests performed under anesthesia remained negative. Examination using the arthrometer such as Kneelax or KT-2000 was not performed. Both bundles of the reconstructed ligament were intact. These results were excellent based on the evaluation criteria defined by Kondo (Figure 6) [13].

**Figure 7:**
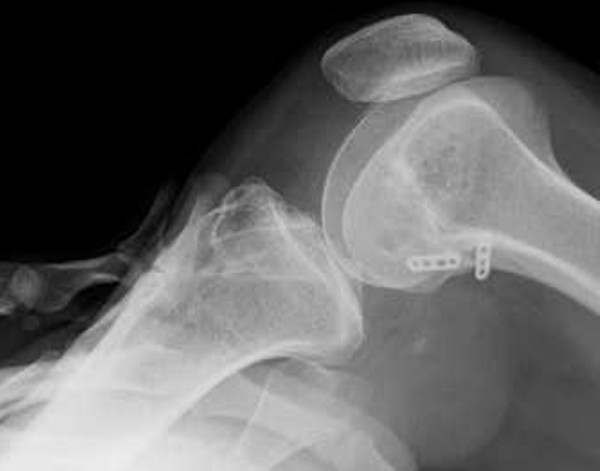
**Stress X-ray findings 36 months after ACL reconstruction. **The results at 36 months post operative stress radiograph evaluation showed 61% anterior translation of the tibia.

The patient examination at 36 months following the ACL reconstruction revealed a Lysholm score of 81 points and an IKDC score of 75.9. The stress radiographic evaluation at 36 months following ACL surgery indicated a 61% anterior translation of the tibia (Figure 7). The patient was able to walk, jog, and ride a bicycle without aid, knee pain, instability or the previous uncomfortable sensation.

## Discussion

The pattern and strength of the involuntary movement associated with athetoid cerebral palsy is variable rather than regular, and becomes more intense at times when the subject is active.Maintaining a constant posture is difficult for this type of patient, and sudden joint movement may occur. Rehabilitation after surgery such as ACL reconstruction can thus be difficult. To date, no reports have described ACL reconstruction in a patient with athetoid cerebral palsy, and appropriate rehabilitation programs for such patients have not been defined. Our patient exhibited pronounced involuntary movement, so we employed cast immobilization for 3 weeks after the ACL reconstruction to inhibit sudden joint movements and stress on the reconstructed ligament. We also slightly restricted extension of the knee by having the patient wear the functional ACL brace thereafter. Full extension of the joint was possible by 6 months after surgery.

Ordinarily, ACL function is evaluated by manual examination or using instruments such as the KT2000 arthrometer and Kneelax. However, in patients with athetoid cerebral palsy, accurate evaluation of knee stability is difficult due to the involuntary movements. Moreover, MRI requires sedation, and cannot be readily repeated due to cost. The patient underwent evaluation by stress radiography, because this technique can be repeated and it is useful as a simple, low-cost modality requiring no special measures.

With the stress radiography technique used, a finding of greater than 70% tibial translation indicates ACL failure [12]. The results of this case demonstrated six month and 36 month postoperative evaluations of 63% and 61% anterior tibial translation, respectively.

If the examiner felt muscle spasticity, he or she repeated the evaluation. The graft was in good condition regarding synovial covering, thickness and tension at the time of the second-look arthroscopy. The stress radiography at this time revealed a 63% translation of the tibia indicating no stretch of the graft. Furthermore, under general anesthesia, the Lachman and pivot shift tests remained negative. These results indicated that the ACL reconstruction was successful in this case.

## Conclusion

This is the first reported case of ACL reconstruction in a patient with athetoid cerebral palsy, with good short-term results. Accurate evaluation of ACL function in such patients is difficult due to the involuntary movements, but stress radiography was useful for evaluating our patient. Anatomical double-bundle autologous hamstring graft ACL reconstruction was performed, and evaluation of the reconstructed ligament revealed good results.

## Consent

Written informed consent was obtained from the patient for publication of this case report and any accompanying images. A copy of the written consent is available for review by the Editor-in-Chief of this journal.

## Abbreviations

ACL: Anterior cruciate ligament; MRI: Magnetic resonance imaging; GMFCS: Gross Motor Function Classification System; IKDC: International Knee Documentation Committee; VAS: Visual analog scale.

## Competing interest

The authors declare that they have no competing interest.

## Authors’ contributions

TT participated in the design of the study and performed the surgery. KY, KK and NY carried out the surgery. EC and SW participated in the design and coordination. All authors read and approved the final manuscript.

## Authors’ information

M.D., Ph.D.

Assistant professor of Division of Orthopedic Surgery, Department of Medicine of Sensory and Motor Organs, Faculty of Medicine, University of Miyazaki.

Member of Japanese Orthopaedics Society.

Member of Japanese Orthopaedics Society for Sports Medicine.

Member of Japanese Society of Clinical Sports Medicine.

Member of Japanese Orthopaedics Society Knee, Arthroscopy and Sports Medicine.

## References

[B1] Dunn WR Lyman S Lincoln AE Amoroso PJ Wickiewicz T Marx RG The effect of anterior cruciate ligament reconstruction on the risk of knee reinjury Am J Sports Med 2004 32 8 1906 1914 10.1177/0363546504265006 15572320

[B2] Granan LP Bahr R Lie SA Engebretsen L Timing of anterior cruciate ligament reconstructive surgery and risk of cartilage lesions and meniscal tears: a cohort study based on the Norwegian National Knee Ligament Registry Am J Sports Med 2009 37 5 955 961 10.1177/0363546508330136 19251674

[B3] Yoo JC Ahn JH Lee SH Yoon YC Increasing incidence of medial meniscal tears in nonoperatively treated anterior cruciate ligament insufficiency patients documented by serial magnetic resonance imaging studies Am J Sports Med 2009 37 8 1478 1483 10.1177/0363546509332432 19359417

[B4] Yasuda K Kondo E Ichiyama H Tanabe Y Tohyama H Clinical Evaluation of Anatomic Double-Bundle Anterior Cruciate Ligament Reconstruction Procedure Using Hamstring Tendon Grafts: Comparisons Among 3 Different Procedure Arthroscopy 2006 22 3 240 251 10.1016/j.arthro.2005.12.017 16517306

[B5] Shino K Nakata K Nakamura N Toritsuka Y Horibe S Nakagawa S Suzuki T Rectangular Tunnel Double-Bundle Anterior Cruciate Ligament Reconstruction with Bone-Patellar Tendon-Bone Graft to Mimic Natural Fiber Arrangement Arthroscopy 2008 24 10 1178 1183 10.1016/j.arthro.2008.06.010 19028171

[B6] Kondo E Yasuda K Azuma H Tanabe Y Tagi T Prospective clinical comparisons of anatomic double-bundle versus single-bundle anterior cruciate ligament reconstruction procedures in 328 consecutive patients Am J Sports Med 2008 36 9 1675 1687 10.1177/0363546508317123 18490472

[B7] Yasuda K Tanabe Y Kondo E Kitamura N Tohyama H Anatomic Double-Bundle Anterior Cruciate Ligament Reconstruction: Current Concepts Arthroscopy 2010 26 9 21 34 10.1016/j.arthro.2010.03.014 20810091

[B8] Bedi A Musahi V O`Laughlin P Maak T Citak M Dixon P Pearle AD A Comparison of the Effect of Central Anatomical Single-Bundle Anterior Cruciate Ligament Reconstruction and Double-Bundle Anterior Cruciate Ligament Reconstruction on Pivot Shift Kinematics Am J Sports Med 2010 38 9 1788 1794 10.1177/0363546510369303 20566720

[B9] Wood E Rosenbaum P The gross motor function classification system for cerebral palsy: a study of reliability and stability over time Dev Med Child Neurol 2000 42 5 292 296 10.1017/S0012162200000529 10855648

[B10] Hefti F Müller W Jakob RP Staubli HU Evaluation of knee ligament injuries with the IKDC form Knee Surg Sports Traumatol Arthrosc 1993 1 226 234 10.1007/BF01560215 8536037

[B11] Yasuda K Kondo E Ichiyama H Kitamura N Tanabe Y Tohyama H Minami A Anatomic reconstruction of the anteromedial and posterolateral bundles of the anterior cruciate ligament using hamstring tendon grafts Arthroscopy 2004 20 10 1015 1025 10.1016/j.arthro.2004.08.010 15592229

[B12] Iseki F Toris T, Kobayashi A Radiographic diagnosis of the knee. Voila Hiza. Volume 3 1994 2 Nankodo, Tokyo 43 55 in Japanese

[B13] Kondo E Yasuda K Second-look arthroscopic evaluations of anatomic double-bundle anterior cruciate ligament reconstruction: relation with postoperative knee stability Arthroscopy 2007 23 11 1198 1209 10.1016/j.arthro.2007.08.019 17986408

